# Reactions of adolescent cyber bystanders toward different victims of cyberbullying: the role of parental rearing behaviors

**DOI:** 10.1186/s40359-024-01879-3

**Published:** 2024-07-04

**Authors:** Qiqi Chen

**Affiliations:** Department of Applied Social Sciences, The Hong Kong Polytechnic University, Hong Kong, China

**Keywords:** Cyberbullying, Bystander, Victimization, Parental rearing, Celebrity

## Abstract

**Background:**

Group-based situations are common settings for cyberbullying, making bystander responses crucial in combating this issue. This study investigated how adolescent bystanders respond to various victims, including family members, friends, teachers, and celebrities. This study also examined how different parenting styles influenced children’s cyber bystander involvement.

**Methods:**

This study employed data from a cross-sectional school survey covering 1,716 adolescents aged 13–18 years from public and vocational schools in China collected in 2022. Logistic regression analyses were conducted to measure demographic characteristics, cyberbullying experiences, and parental rearing behaviors in predicting bystander reactions.

**Results:**

The findings showed that middle school students preferred to “ask for help” while high school students tended to choose “call the police” when witnessing cyberbullying incidents. Bystanders growing up with parental rejection and overprotection, having previous cyberbullying victimization experiences, where the victims were disliked by them, exhibited fewer defensive reactions.

**Conclusions:**

This study has implications for future research and practices involving parental involvement in cyber bystander interventions, which could provide implications for future practice in designing specific intervention programs for cyberbullying bystander behavior. Future research and interventions against cyberbullying may provide individualized training including parents’ positive parenting skills and parent-child interactions.

## Background

### Cyberbullying and health correlates

The increased accessibility of digital technology and online communication has created both opportunities and risks for young people as they navigate the online world [1]. Cyberbullying refers to the intentional and repetitive harm inflicted through electronic devices, such as computers or cell phones [2]. The anonymity of perpetrators and the ease of disseminating information in cyberspace distinguish cyberbullying from traditional bullying in many ways [2, 3]. Consequently, victims of cyberbullying often face higher risks of psychological problems than victims of traditional bullying, such as loneliness, reduced self-efficacy, depression, lower self-esteem, anti-social behavior, and even suicide attempts [2, 4]. During adolescence, individuals are developing their identities and are more sensitive to peer influence and opinions, which can make negative online interactions, such as cyberbullying, particularly hurtful and damaging. Studies have indicated that cyberbullying is most prevalent among adolescents [3], most commonly observed during early to mid-adolescence, between the ages of 12 and 15 years, and its incidence rates tend to increase during secondary education [5]. Older students, often more digitally proficient, may spend more time on a wider range of online platforms. This can affect the likelihood of encountering cyberbullying and may lead to different engagement in or witnessing of cyberbullying compared to younger students [5]. Although most adolescents exposed to cyberbullying are not directly involved in these behaviors, their inactivity can have negative consequences on their mental health and physical functioning [6]. Stress and negative emotions are common responses to witnessing cyberbullying [7]. The stress response may be due to a perceived or actual lack of power to stop the bullying, feelings of helplessness, or concerns about becoming the next victim [8]. Therefore, intervention and prevention programs addressing bystander involvement in cyberbullying are crucial.

### The role of cyberbullying bystanders

Individuals who witness a victim being bullied online, also known as bystanders, play a crucial role in cyberbullying dynamics [9, 10]. The Bystander Effect Framework proposes that individuals go through five key stages when considering intervening to help someone [11]: noticing the situation, recognizing the assistance needed, feeling a sense of responsibility to intervene, believing that they have the skills, and deciding to intervene. Bystanders can choose to remain silent, providing no support to either the bully or the victim [12]. The silence of witnesses, often due to fear of retaliation or peer rejection, can be perceived as a form of acceptance of the perpetration that contributes to continued perpetration and victimization [4]. Individual characteristics may play a role in different bystander reactions, where students in higher grades may have a more sophisticated understanding of the consequences of their actions online through education [9, 10]. The social hierarchies and peer relationships that evolve with each grade level can also influence awareness of cyberbullying behaviors and the likelihood of engaging in positive bystander behavior [11, 12]. Bystander intervention can be effective in school bullying, as perpetrators often seek peer approval, which can manifest as a tacit endorsement of their aggressive behavior [6]. Similarly, bystander reactions in online settings can either reinforce or mitigate harmful behavior through actions like clicking a button to “like” or “retweet” on social networking sites, combined with a diffusion of responsibility in large online audiences [8, 10]. Research has shown that bystanders are less likely to intervene in online contexts than in face-to-face bullying incidents because of the physical distance between the victim and bystanders, making it difficult for bystanders to directly witness the victim’s suffering and assess the severity of the incident [13]. Visual anonymity in the online environment might also decrease the likelihood of bystanders acting when they perceive the incident as less serious, thus leading to victim blaming [14]. Furthermore, the absence of clear rules and regulations in cyberspace can create ambiguity, leading bystanders to ignore incidents rather than intervene.

### Parental rearing behaviors and cyber-bystander reactions

Families that provide affection, trust, and open communication reduce the likelihood of risky situations such as bullying [15]. Parental rearing shapes the foundational attitudes, values, and behaviors of children. Parental Mediation Theory focuses on how parents intervene in their children’s media use, which suggests that parental strategies can influence children’s media experiences and their reactions to content [16, 17]. Effective parental mediation is a combination of responsibilities, including restrictive measures (e.g., rule setting), active communication about media use, and co-viewing activities (e.g., watching television together) [18]. These strategies aim to promote informed, responsible, and critical media consumption while safeguarding adolescents from potential online risks. Children raised by supportive parents are likely to mirror the supportive experiences from home and defend victims in bullying situations when they are bystanders [19]. To effectively guide and serve as role models for their children, parents need to possess sufficient knowledge and a sense of perceived responsibility that enables them to communicate effectively and protect their children from potential risks. Specifically, cultivating instructive mediation in the context of cyberbullying involves developing a solid understanding of which behaviors can be considered cyberbullying and which fall outside of that definition [20]. Accurately identifying online misbehaviors enables parents to be aware of the virtual environment in which their children interact daily and is crucial for providing appropriate guidance and better support to their children [21].

Parents are also advised to employ conflicting strategies, including maintainging open and honest communication with their children about coping strategies for cyberbullying, as well as monitoring children’s use of digital technology [22]. Studies have reported that adolescents are less likely to engage in cyberbullying, either as victims or perpetrators, if their parents provide effective online supervision and monitoring [23, 24]. In contrast, victims of cyberbullying often come from disadvantaged or violent families with limited family social capital [25]. The complexities of digital technologies sometimes generate confusion and concern for parents [26]. Many parents acknowledge that their children are more adept at technology and may find ways to circumvent these restrictions [27]. Parents may feel ambivalent about their children’s access to technology, viewing monitoring or restricting children’s use of digital technology as good parenting behavior that can protect children from cyberbullying [23]. Therefore, some adolescents do not report cyberbullying victimization or incidents they witness to adults due to several reasons: they feel that parents cannot help, they do not want to burden their parents, they perceive that teachers do not react to cyberbullying incidents, or they prefer to solve problems by themselves [28]. Thus, effective parental rearing involves teaching children social skills that are often critical in determining whether a child becomes an active bystander or remains passive.

### Factors to different bystander reactions

Knowledge of how bystanders react to different cyberbullying victims can inform the development of prevention strategies that encourage positive bystander behavior, such as reporting the bullying or supporting the victim. Individual responses can reflect underlying social norms and attitudes toward different groups of people. Parental Mediation Theory also illustrates how different parental mediation strategies influence children’s reactions as cyber-bystanders to various victims of cyberbullying. Discussions about social norms and in-group biases further shape these reactions, making children more or less likely to intervene based on the victim’s perceived similarity to their own social or cultural group. Active content mediation fosters a deeper understanding and consistent intervention across different scenarios, whereas restrictive mediation may limit children’s exposure to diversity, thereby reducing their likelihood to act in unfamiliar situations. Collectively, these mediation strategies profoundly impact how children perceive and react to cyberbullying incidents, influencing their actions as bystanders in the digital world. For example, gender socialization can impact the level of empathy adolescents show toward victims, where girls are often socialized to be more empathetic and nurturing, which might make them more likely to offer emotional support to victims [9, 10]. Scholars have suggested that bystanders’ motivations and activation of empathy differ based on the victim’s identity [15]. Bystanders are unlikely to perceive celebrity victims as weak because they have a substantial fan base and may consider negative comments part of the celebrity’s job description [29]. Evidence shows that 14% of bystanders engage in offensive commenting or posting about celebrity victims [9]. In comparison, researchers have found that when the social networks of the bystander overlap with those of the victims, such as if the victims are family members and friends, expected social capital gains could influence a bystander’s relational investment in cyberbullying events [30]. Individuals witnessing such cyberbullying incidents often have real-life connections, which means that their responses can have consequences for their offline impact on victims [31]. By contrast, if bystanders become deindividualized, disinhibited, or feel a diminished sense of responsibility for their online actions, then the online environment may cause them to actively support perpetrators because of their desire to affiliate with the stronger group [4, 32]. These supportive reactions could take the form of forwarding or sharing hurtful messages or reinforcing the perpetrator’s posts in their own private messages or posts [32]. The re-sharing of aggressive messages may amplify cyberbullying to extreme proportions [33]. Instructive mediation, where parents discuss the impacts of bullying and the importance of empathy, can encourage children to support victims more actively, although the level of support may vary depending on the specific emphasis of parental guidance [16, 17]. When parents model behavior, they inadvertently teach children whom to empathize with, possibly leading to biased bystander behaviors [20]. Therefore, it is necessary to investigate the parenting factors of bystanders’ different evaluation logics regarding their involvement with different victims. Understanding how individuals respond to various cyberbullying scenarios can help in creating tailored interventions that address specific types of victimization.

### Current study

The review above underscores the significant role of parental influence on children’s behavior online. It suggests that engaged parents who communicate openly about online activities can cultivate children who are more thoughtful and effective in their responses to cyberbullying and other online challenges. By emphasizing on parental rearing, interventions can be designed to prevent negative bystander behavior before it starts, rather than merely addressing it after the fact. Early intervention through parenting education programs is recognized as a crucial trategy for preventing child victimization [34]. Globally, a variety of parenting education programs have been implemented, including the Systematic Training for Effective Parenting (STEP) program and the Positive Parenting Programme (Triple P) [35]. The outcomes of these programs have been significant, showing improvements in parenting skills and reductions in child victimization [36]. Additionally, proficient parenting skills act as a protective mechanism, reducing the risk of children facing mental health challenges [37]. While many parenting education programs focus on conveying specific information and skills, they often overlook critical aspects of interventions for parents concerning cyber-bystander behaviors. This indicates a need for further investigation into how parental influences support their children and promote positive bystander behaviors in online environments. Thus, this study aimed to explore different cyber-bystander reactions to various cyberbullying victims, with a special focus on the role of parental rearing behaviors. We hypothesized that (H1) adolescents will choose different strategies in response to witnessed cyberbullying incidents involving various victims, and (H2) different parental rearing styles are associated with different cyber-bystander behaviors.

## Methods

### Study design and procedure

This study employed data from a school survey conducted with students in grade 8–12, aged 13–18 years, in China from September to October 2022. Two public schools in Qingdao and one high school in Wuhan were selected through a convenient sampling method. The study aimed to capture adolescent cyber bystander behaviors in each of these two cities. All classes in the selected schools were invited to participate. Eligible students included all full-time students at these schools, irrespective of gender, family socioeconomic status, birthplace, academic performance, migration status, or relationships with their caregivers. Students who received parental consent were verbally informed about the objectives and content of the study, as well as their right to withdraw at any time without penalty. Those willing to participate signed an informed consent form and also provided parental consent from their legal guardians before participating in the survey. These students were then invited to complete a web-based questionnaire under the instructions of trained research assistants. The survey took approximately 30 min to complete. In total, 1,716 adolescents participated in the study. The average age of the participants was 14.60 years (SD = 1.35), and over half of the participants (55.48%, *n* = 952) were female. Ethical approval for the study was granted by the Institutional Review Board of the authors’ affiliated university.

### Measures

#### Cyberbullying experiences

Participants’ own cyberbullying experiences in the preceding year were measured with the 14-item Chinese version of the European Cyberbullying Intervention Project Questionnaire (ECIPQ-C) [38, 39]. The ECIPQ-C contains seven items each on cyberbullying perpetration and victimization. Example items were “I excluded or neglected someone in a social networking site” and “I was excluded or ignored by someone in a social networking site or Internet chat room.” Each item is rated on a 5-point Likert scale (0 = “never,” 4 = “always”). We then recoded the cyberbullying experiences into three clusters: perpetration, victimization, and perpetration-victimization [40], where those who reported at least one item as 1 in both the perpetration and victimization subscales were grouped into “Perpetration-victimization.” The reliability of the total scale was good with Cronbach’s alpha of 0.96. The two subscales also showed good reliability, with Cronbach’s alpha for perpetration and victimization being 0.98 and 0.93, respectively.

#### Bystander reactions

Several possible reactions as bystanders of cyberbullying were constructed by referring to the categories of problem-focused coping, emotion-focused coping, and avoidant coping in previous literature [41]. Bystander reactions to cyberbullying were assessed by nine items, including “wait and see,” “seek the truth,” “retaliate online,” “forward and spread,” “tip off,” “ask for help,” “call the police,” and “pay no attention.” The participants’ perceived reactions as bystanders towards different cyberbullying victims were assessed based on their roles and subjective attitudes towards the victims. Specifically, victims could be family members, liked schoolmates, disliked schoolmates, liked teachers, disliked teachers, liked public figure, or disliked public figure. The items were multiple-selective, asking participants to check all the responses that applied to them. The details of all reaction behaviors are listed in Table 1. For descriptive analysis, all of the above items were multiple-choice options for participants to select if applicable in their cases. The results were recoded as 1 (yes) if participants selected such case and as 0 if they chose “never.”

**Table 1:** Cyberbullying, bystander reactions, and parental rearing behaviors by grade

*N* (%)	Total (*N* = 1,716)	Middle 2 (*N* = 587)	Middle 3 (*N* = 527)	High 1 (*N* = 231)	High 2 (*N* = 223)	High 3 (*N* = 148)	*p*-value^a^
Age, M (SD)	14.60 (1.35)	13.36 (0.57)	14.39 (0.58)	15.15 (0.87)	16.12 (0.60)	17.08 (0.73)	183.60***
Gender							21.36***
Boy	764 (44.52)	304 (51.79)	222 (42.13)	88 (38.10)	85 (38.12)	65 (43.92)	
Girl	952 (55.48)	283 (48.21)	305 (57.87)	143 (61.90)	138 (61.88)	83 (56.08)	
Cyberbullying							
Perpetration	216 (12.59)	29 (4.94)	37 (7.02)	54 (23.38)	61 (27.35)	35 (23.65)	131.13***
Victimization	610 (35.55)	98 (16.70)	151 (28.65)	142 (61.47)	133 (59.64)	86 (58.11)	259.13***
Perpetration-victimization	208 (12.12)	27 (4.60)	32 (6.07)	53 (22.94)	61 (27.35)	35 (23.65)	141.72***
Bystanders’ reactions							
Wait and see	625 (36.42)	159 (27.09)	213 (40.42)	97 (41.99)	99 (44.39)	57 (38.51)	35.22***
Seek the truth	911 (53.09)	294 (50.09)	316 (59.96)	125 (54.11)	110 (49.33)	66 (44.59)	17.78**
Retaliate online	314 (18.30)	74 (12.61)	121 (22.96)	50 (21.65)	39 (17.49)	30 (20.27)	22.59***
Forward and spread	240 (13.99)	54 (9.20)	92 (17.46)	38 (16.45)	29 (13.00)	27 (18.24)	20.03***
Tip off	1,063 (61.95)	353 (60.14)	365 (69.26)	142 (61.47)	127 (56.95)	76 (51.35)	22.21***
Ask for help	1,139 (66.38)	462 (78.71)	337 (63.95)	139 (60.17)	121 (54.26)	80 (54.05)	70.09***
Call the police	1,246 (72.61)	453 (77.17)	395 (74.95)	156 (67.53)	141 (63.23)	101 (68.24)	21.88***
Pay no attention	415 (24.18)	122 (20.78)	116 (22.01)	73 (31.60)	60 (26.91)	44 (29.73)	15.37**
Parental rearing behaviors, M (SD)							
Reject	10.60 (4.07)	9.70 (3.67)	10.69 (4.35)	11.62 (4.10)	11.46 (4.00)	10.90 (3.96)	41.55***
Warmth	19.78 (5.21)	21.57 (4.51)	20.45 (4.97)	17.62 (5.30)	17.04 (5.02)	17.84 (5.35)	103.94***
Overprotection	17.85 (4.41)	17.86 (4.01)	18.44 (4.46)	17.67 (4.64)	17.10 (4.84)	17.09 (4.46)	104.02***

Note. *p < 0.05. **p < 0.01. ***p < 0.001

^a^P-value by X^2^ test or t-test

#### Parental rearing behaviors

Parental rearing behaviors were measured using the 21-item short-form Egna Minnen and Barndoms Uppfostran for the Chinese scale (s-EMBU-C) [42]. The scale included three factors: rejection (six items), emotional warmth (seven items), and overprotection (eight items). Example items are “My parents get angry with me without letting me know the reason,” “My parents try to encourage me to become the best,” and “My parents get overly anxious that something might happen to me” respectively. Each item is rated on a 4-point Likert scale (1 = “never,” 4 = “always”). The reliability of the total scale was good, with Cronbach’s alpha of 0.87. The three subscales showed satisfactory reliabilities with rejection (0.89), emotional warmth (0.92), and overprotection (0.76).

### Statistical analysis

Demographic characteristics and the prevalence of cyberbullying experiences were summarized using a descriptive analysis. The prevalence of bystander reactions and parental rearing behaviors was computed using descriptive statistics and divided by grade. The above literature review suggests that cyberbullying victimization and the role of bystanders may differ significantly across developmental stages. By categorizing the sample into grades, we aim to explore how these differences manifest during different stages of adolescence. Differences in the above characteristics according to grade were evaluated using *t*-tests and chi-square tests. The percentages of different bystander reactions were grouped and compared based on the role of the cyberbullying victims. Pearson’s correlation analysis was performed to analyze the relationships among all outcome measures and compared by gender. Finally, logistic regression analyses were conducted to measure demographic characteristics, cyberbullying experiences, and parental rearing behaviors in predicting bystander reactions. The level of significance was set at *p* < 0.05. Stata version 17.0 was used to perform all analyses.

## Results

### Descriptive statistics of outcome measures by grade

Participants’ cyberbullying experiences, bystander reactions, and parental rearing behaviors were summarized by grade as shown in Table 2. A total of 610 (35.55%) participants reported cyberbullying victimization experiences and 216 (12.59%) reported cyberbullying perpetration. A generally increasing trend was found in cyberbullying experiences, among which high school students reported significantly higher rates of cyberbullying perpetration and victimization than middle school students (all *p* < 0.001). The most frequently chosen approaches for dealing with cyberbullying as bystanders were to “call the police” (72.61%), “ask for help” (66.38%), and “tip-off” (61.95%). Passive reactions such as “forward and spread” (13.99%) and “retaliate online” (18.30%) appeared to be the least preferred. We also noticed that the most prevalent approach was to “ask for help” for middle school students (63.95 to 78.71%) and “call the police” for high school students (63.23 to 68.24%). A generally increasing trend along with grades was found for parental warmth and overprotection, whereas a decreasing trend was found for parental warmth.

**Table 2:** Bystander reactions by cyberbullying victims

*N* (%)	Family member	Schoolmate-Like	Schoolmate-Dislike	Teacher-Like	Teacher-Dislike	Celebrity-Like	Celebrity-Dislike
Wait and see	284 (16.55)	326 (19.00)	398 (23.19)	324 (18.88)	384 (22.38)	389 (22.67)	439 (25.58)
Seek the truth	730 (42.45)	677 (39.45)	541 (31.53)	680 (39.63)	556 (32.40)	633 (36.89)	494 (28.79)
Retaliate online	191 (11.13)	167 (9.73)	99 (5.77)	126 (7.34)	88 (5.13)	163 (9.50)	88 (5.13)
Forward and spread	112 (6.53)	109 (6.35)	82 (4.78)	86 (5.01)	85 (4.95)	128 (7.46)	93 (5.42)
Tip off	927 (54.02)	867 (50.52)	696 (40.56)	839 (48.89)	701 (40.85)	718 (41.84)	632 (36.83)
Ask for help	771 (44.93)	1,021 (59.50)	806 (46.97)	749 (43.65)	620 (36.13)	527 (30.71)	492 (28.67)
Call the police	1,170 (68.18)	963 (56.12)	822 (47.90)	1,016 (59.21)	841 (49.01)	822 (47.90)	746 (43.47)
Pay no attention	86 (5.01)	102 (5.94)	239 (13.93)	105 (6.12)	232 (13.52)	232 (13.52)	339 (19.76)

### Bystander reactions by cyberbullying victims

Participants’ bystander reactions were categorized and compared among victims in the witnessed cyberbullying situation. Overall, the most selected bystander reactions for specific victims were: “call the police” (68.18%) and “tip off” (54.02%) for family members; “ask for help” (59.50%) and “call the police” (56.12%) for liked schoolmate; “call the police” (59.21%) and “tip off” (48.89%) for liked teacher; “call the police” (47.90%) and “tip off” (41.84%) for liked celebrity. Participants reported lower rates of positive bystander reactions for disliked people. For example, the highest rates of reaction approaches for disliked celebrity were “wait and see” (25.58%) and “pay no attention” (19.76%), while those for family members were “seek the truth” (42.45%) and “retaliate online” (11.13%).

### Correlations among outcome variables by gender

As shown in Table 3, the correlations between bystanders’ reactions and parental rearing behaviors are summarized and presented by gender. Among boys, we found that parental rejection was positively related to passive reactions such as “wait and see” (*r* = 0.08, *p* < 0.05), “retaliate online” (*r* = 0.13, *p* < 0.001), “forward and spread” (*r* = 0.14, *p* < 0.001), and “pay no attention” (*r* = 0.16, *p* < 0.001). Parental rejection was negatively related to positive bystander reactions, including “ask for help” (*r* = -0.15, *p* < 0.001) and “call the police” (*r* = -0.14, *p* < 0.001). Parental warmth was positively related to positive bystander reactions (*r*s ranged from 0.13 to 0.26, all *p*s < 0.001) and negatively related to passive reactions (*r*s ranged from − 0.15 to -0.15, all *p*s < 0.001). Parental overprotection was only found positively related to passive reactions (*r*s ranged from 0.08 to 0.08, all *p*s < 0.05). Similar patterns were found among girls, except that parental warmth was positively related to “tip-off” (*r* = 0.12, *p* < 0.001). Relatively greater correlation coefficients between parental rearing behaviors and bystander reactions were reported among boys than girls.

**Table 3:** Correlations matrix of bystander reactions and parental rearing behaviors by gender

	Boy/girl	1	2	3	4	5	6	7	8	9	10	11
1	Parenting-reject	/	-0.29***	0.71***	0.10**	-0.03	0.10**	0.09**	-0.05	-0.14***	-0.17***	0.13***
2	Parenting-warmth	-0.08*	/	0.16***	-0.16***	0.08*	-0.05	-0.03	0.12***	0.20***	0.17***	-0.09**
3	Parenting-overprotection	0.73***	0.32***	/	-0.01	0.01	0.10**	0.08*	0.02	-0.02	-0.05	0.05
4	Wait and see	0.08*	-0.15***	-0.02	/	0.10**	0.16***	0.12***	-0.13***	-0.21***	-0.30***	0.09**
5	Seek the truth	-0.07	0.13***	0.03	0.09*	/	0.24***	0.20***	0.36***	0.24***	0.17***	-0.12***
6	Retaliate online	0.13***	-0.04	0.08*	0.16***	0.23***	/	0.47***	0.19***	0.01	0.01	0.08*
7	Forward and spread	0.14***	-0.04	0.08*	0.14***	0.16***	0.57***	/	0.16***	0.06	0.07*	0.06
8	Tip off	-0.01	0.07	0.05	-0.17***	0.33***	0.23***	0.17***	/	0.24***	0.35***	-0.24***
9	Ask for help	-0.15***	0.26***	-0.01	-0.22***	0.29***	0.07*	0.08*	0.29***	/	0.40***	-0.25***
10	Call the police	-0.14***	0.23***	-0.01	-0.30***	0.17***	0.04	0.04	0.31***	0.39***	/	-0.34***
11	Pay no attention	0.16***	-0.15***	0.08*	-0.03	-0.16***	0.07	0.07*	-0.18***	-0.23***	-0.27***	/

Note. *p < 0.05. **p < 0.01. ***p < 0.001

### Regression analysis among demographic and outcome measures

To further examine the effects of gender, grade, cyberbullying, and parental rearing behaviors on bystander reactions, we conducted logistic regression analyses (Table 4). We found that girls were more likely to take actions including both positive and passive approaches (*B*s range from 0.22 to 0.60, *p*s < 0.05) than boys did. Participants in higher grades reported more “wait and see” (*B* = 0.09, *p* < 0.05), but less “tip off” (*B* = -0.13, *p* < 0.01) and “ask for help” (*B* = -0.20, *p* < 0.001) approaches. Cyberbullying victimization experiences had a positive effect on self-reliant cyber bystander behaviors such as “seek the truth” (*B* = 0.74, *p* < 0.001), “retaliate online” (*B* = 0.73, *p* < 0.001), “forward and spread” (*B* = 0.45, *p* < 0.05), and “tip off” (*B* = 0.69, *p* < 0.001). Notably, the dual roles of cyberbullying perpetration-victimization had a significantly negative effect on “wait and see” (*B* = -1.75, *p* < 0.05), which was the largest effect size among others. No significant relationship was found between cyberbullying perpetration and bystander reaction.

**Table 4:** Logistic regression of gender, cyberbullying, parental rearing behaviors, and bystander reactions

B (SE)	Wait and see	Seek the truth	Retaliate online	Forward and spread	Tip off	Ask for help	Call the police	Pay no attention
Gender ^a^	-0.03 (0.10)	0.23* (0.10)	0.28* (0.13)	0.60*** (0.15)	0.22* (0.10)	0.53*** (0.11)	0.52*** (0.11)	-0.06 (0.12)
Grade	0.09* (0.04)	-0.06 (0.04)	-0.01 (0.05)	-0.01 (0.06)	-0.13** (0.04)	-0.20*** (0.04)	-0.06 (0.05)	0.06 (0.05)
Cyber-perpetration	1.11 (0.74)	-0.06 (0.71)	0.48 (0.85)	0.79 (0.84)	-0.96 (0.74)	-0.28 (0.75)	0.02 (0.83)	1.25 (0.72)
Cyber-victimization	0.33 (0.13)	0.74*** (0.13)	0.73*** (0.15)	0.45* (0.17)	0.69*** (0.14)	0.13 (0.14)	0.35* (0.15)	0.19 (0.14)
Cyber Perpetration-victimization	-1.75* (0.77)	-1.13 (0.74)	-0.78 (0.87)	-0.51 (0.87)	0.17 (0.76)	-0.42 (0.77)	-0.99 (0.85)	-1.09 (0.75)
Parenting-reject	0.05* (0.02)	-0.02 (0.02)	-0.02 (0.03)	0.03 (0.03)	-0.01 (0.02)	-0.05 (0.02)	-0.09*** (0.02)	0.02 (0.02)
Parenting-warmth	-0.05 (0.01)	0.01 (0.01)	-0.05 (0.02)	-0.05** (0.02)	-0.01 (0.01)	0.06*** (0.01)	0.05*** (0.01)	-0.04 (0.01)
Parenting-overprotection	-0.01 (0.02)	0.04 (0.02)	0.08** (0.03)	0.05 (0.03)	0.02 (0.02)	0.02 (0.02)	0.05* (0.02)	0.03 (0.02)
R-square	0.04	0.03	0.05	0.05	0.02	0.07	0.07	0.03
p	< 0.001	< 0.001	< 0.001	< 0.001	< 0.001	< 0.001	< 0.001	< 0.001

Note. *p < 0.05. **p < 0.01. ***p < 0.001

^a^ Gender: Boys = 1, girls = 2

Regarding parental rearing behaviors, we found that parental rejection had a positive effect on “call the police” (*B* = -0.09, *p* < 0.001), and a negative effect on “wait and see” (*B* = 0.05, *p* < 0.05). Parental warmth had a positive effect on “ask for help” (*B* = 0.06, *p* < 0.001) and “call the police” (*B* = 0.05, *p* < 0.001), and a negative effect on “forward and spread” (*B* = -0.05, *p* < 0.01). Parental overprotection had a positive effect on “retaliate online” (*B* = 0.08, *p* < 0.01) and “call the police” (*B* = 0.05, *p* < 0.05).

## Discussion

Bystanders play a critical role in cyberbullying incidents, yet there is still much to understand about the factors influencing their responses. While previous research has identified individual factors that predict bystanders’ defensive reactions to cyberbullying [33], the significant role of parental involvement in adolescent development necessities the inclusion of parenting factors in these explorations. This study investigated various forms of cyber bystander reactions to different cyberbullying victims, including asking for help, calling the police, retaliating online, and paying no attention. Our findings also revealed notable differences in reactions between individuals with and without personal experiences of cyberbullying. Additionally, the study highlighted how various parental rearing styles impact adolescents’ responses as cyber bystanders. By examining reactions to different types of cyberbullying victims, our approach not only deepened the understanding of bystander reactions but also enhanced the generalizability of the findings. These insights could be instrumental in designing targeted intervention programs at improving cyberbullying bystander interventions.

The results revealed a discernible increase in both cyberbullying perpetration and victimization from middle to high school, with high school students reporting more cyberbullying incidents than middle school students. Notably, middle school students tended to prefer “ask for help” when witnessing cyberbullying, while high school students tended to choose “call the police”. We observed higher rates of cyberbullying victimization among students in Middle 2, which could be attributed to early adolescence. During this period, emotional and physiological changes can heighten sensitivity to peer relationships and negative interactions, such as bullying. Additionally, increased access to digital devices, reduced parental supervision, and the emulation of aggressive behaviors by older students—who often go unaddressed—may further expose younger students to cyberbullying. While younger students may lack the skills and knowledge to intervene effectively, they tend to seek help [6]. Older students, however, may choose to take self-reliant actions, such as calling the police and retaliating online [43]. This behavior could be partly explained by the online disinhibition effect, where the anonymity of the online environment emboldens some individuals to act in ways they would not consider offline [44]. These findings underscore the importance of schools to implementing tailored initiatives within their cyberbullying prevention curricula. By encouraging active bystander strategies and fostering constructive intervention approaches, these programs can be adapted to meet the needs of different grade levels. For example, allowing older students to share their successful intervention strategies with younger peers could promote a range of constructive approaches to handling cyberbullying, whether as victims of bystanders. This approach not only enriches the learning experience but also leverages the insights gained from the study to enhance school-based cyberbullying interventions, using grade levels as a framework to categorize and structure these initiatives.

We found that participants tend to exhibit more passive reactions, such as “wait and see” and “pay no attention”, when the victims are disliked celebrities. Conversely, more active and defensive reactions such as “seek the truth” and “retaliate online” are prominent when family member are the victims. While previous research has emphasized the role of empathy in activating bystanders to combat cyberbullying [30], our study suggests the need to consider how bystanders’ evaluation logics differ based on the victim’s identity. Research has shown that larger audience sizes and the public nature of cyberspace might inhibit bystanders’ pro-celebrity behaviors, possibly due to the fears of retaliation from perpetrators or their supporters [45]. Moral Disengagement Theory suggests that bystanders may expect others to intervene, thus decreasing the likelihood that individual action. This inaction may be perceived as increasing the risk of retaliation in cases of celebrity cyberbullying. Additionally, societal hierarchies may deter intervention when high-status individuals are involved, as bystanders might assume these individuals have sufficient legal and social resources to defend themselves. However, when cyberbullying targets family members and friends, cultural norms that discourage intervening in what are seen as private matters can also inhibit bystander intervention. These norms can lead to a reluctance to take action, even against public displays of negativity or harassment toward family members online. Considering the large scale of celebrity cyberbullying, understanding the factors that influence bystanders’ willingness to help celebrities is crucial [31, 46]. For example, fans who feel a personal connection to a celebrity may be more inclined to defend them. Platform owners have a role to play here; they can initiate campaigns against celebrity cyberbullying to mitigate its negative effects and promote positive bystander behavior.

The findings of this study showed that participants who experienced parental rejection and overprotection reported more passive bystander reactions, whereas those with more parental warmth tended to choose positive bystander reactions. Notably, adolescents who benefited from greater parental warmth, particularly girls, often displayed proactive defenses such as reporting cyberbullying incidents. Previous research has emphasized the crucial role of parental awareness of online risks and active supervision in preventing cyberbullying [47]. Our findings support this, showing that consistent emotional and practical parental support can empower children to actively oppose cyberbullying. However, overprotective parenting, which limits children’s autonomy, and excessively controlling behaviors can hinder the development of a child’s ability to independently assess and respond in bystander situations. While many parents respect their children’s agency in cyberspace and provide considerable freedom, they tend to intervene only when situations become critical [24]. The validation provided by parents, reinforcing the child’s self-worth and moral values, appears to influence the likelihood of a child intervening in cyberbullying scenarios positively. However, the challenge remains that many parents lack the necessary knowledge, perceive themselves as incompetent, or are unaware of the risks associated with cyberbullying, which hampers their ability to prevent or address such incidents effectively [15]. This highlights the need for enhanced awareness and co-responsibility between families and schools to collaborate more closely in supporting and empowering children to combat cyberbullying effectively [48].

Our study found that individuals who had experienced cyberbullying victimization tended to choose more self-reliant bystander behaviors, such as seeking truth and retaliating online, whereas whose who had been both perpetrators and victims of cyberbullying (perpetrator-victims) were least likely to adopt a “wait and see” approach when witnessing cyberbullying incidents. This increased proactivity may stem from their heightened awareness of the consequences of cyberbullying, influenced by their personal experiences. This is consistent with previous findings that some children are hesitant to seek help from teachers or parents because they perceive that elders lack the necessary skills and confidence to effectively address cyberbullying [49]. Furthermore, individuals without personal cyberbullying experiences are more likely to refrain from intervening [50]. Moral Disengagement Theory explained this by a distortion of consequences that, unlike the the bystander effect observed in real-life aggression, where bystander may only become aware of online incidents after they have occurred [33]. Individuals with previous trauma could experience higher levels of concern about not intervening and potentially regret not doing so for victims [8]. Individuals with a history of being both cyberbullying victims and perpetrators might experience increased empathy towards victims, possibly reducing their likelihood of adopting a passive stance. Their previous involvement in cyberbullying could also provide them with insights into effective intervention strategies and a sense of responsibility to prevent further instances. This insight likely influences their belief that formal interventions might be inadequate once bullying has occurred, prompting more immediate action when they witness cyberbullying. Anti-bullying policies can take the form of peer support services, which are helpful in supporting victims [44]. Schools may encourage students to have open discussions with teachers and parents about cyberbullying, disclosing not only their own victimization but also seeking help from others.

### Limitations

It is important to consider the limitations of this study when interpreting the results. First, the self-report design for collecting information on cyberbullying and bystander behavior could be affected by response bias and social desirability. There is a possibility that participants reported a higher agreement with positive bystander intentions despite not reflecting their actual behavior in real life. Future studies should include multiple informants or experimental designs for more objective data collection. Second, we examined only individual attitudes toward helping different victims, and the absence of social and contextual factors may have unintentionally introduced ambiguity into the conditions of cyberbullying. Future research may replicate our findings on various social media platforms that enable an examination of the effectiveness of bystander behaviors in multiple settings. Third, the sample from Qingdao and Wuhan obtained through convenience sampling may not be fully representative due to the limited number of schools, the focus on new first-tier cities, and the lack of diversity in school types. Future research should include a wider range of schools across different regions and a random sampling method to better reflect the broader population of adolescents in China. Fourth, in our analysis, the coefficients associated with parental rearing behaviors were relatively smaller compared to other factors such as cyber victimization, gender, and grade. This observation might suggest a less immediate impact of parental behaviors on the dynamics of cyberbullying compared to more direct demographic factors. However, it is crucial to consider that the influence of parental behaviors might manifest more subtly and over a longer term. Future research could benefit from employing longitudinal designs and statistical methods such as path analysis to better capture these nuanced effects. Additionally, expanding the study to include qualitative data could provide deeper insights into how parental influences shape adolescent behaviors in online settings.

### Implications

By enhancing our understanding of cyber bystander behavior, our research provides valuable insights into how to effectively intervene in cyberbullying towards different victims on social media. First, promoting constructive victim-focused bystander intervention responses [51, 52] may help adolescents reflect on the various actions of standers and discuss the outcomes of their bystander role. Future research could integrate these response dimensions with various cultural factors and broader social contexts, such as the critical components of cyberbullying incidents, the efficacy of teacher interventions, and the influence of bystander diffusion of responsibility. Regarding schoolmates, victims who are perceived as relatable or similar to the bystander may elicit stronger empathetic responses and garner more support. Conversely, bystanders might be more inclined to blame victims they dislike or view as having contributed to their own predicament, reducing the likelihood of intervention. School staff and policymakers should cultivate a positive training curriculum for students to recognize the importance of constructive responses to assist different victims. Social media platforms could develop more nuanced reporting mechanisms that are easily accessible and provide bystanders with multiple options for reporting harmful content. For example, online forums could implement educational pop-ups that inform bystanders about the consequences of their actions and encourage positive interventions. Instant messaging software could incorporate interface designs that facilitate the use of supportive button reactions, enabling quick and supportive responses from bystanders. Short video platforms could streamline their reporting processes to acknowledge bystander interventions more effectively.

Second, the findings from this study underscore the importance of nurturing an empathetic and proactive stance in children towards cyberbullying, facilitated by effective parental guidance and involvement. The potential for parental neglect may also create an emotional void that diminishes the child’s propensity to empathize with victims, reducing the impulse to act in defense of others. These findings could be used by future intervention programs to expand parental mediation, deepen their understanding of online relationships and associated risks, and take responsibility for educating their children on victim- and bystander-based reaction behaviors. Educational initiatives targeting parents could be developed to enhance their understanding of cyberbullying dynamics and the critical role they play as mediators. These programs should aim to equip parents with the skills to discuss sensitive online issues, recognize signs of distress in their children, and intervene appropriately. Intervention programs could be designed to involve entire families, helping to bridge any potential emotional voids that might exist between parents and children. These interventions can focus on fostering open communication channels within the family, where children feel comfortable discussing their online experiences and bystander encounters. Future research may explore in greater depth how different parenting styles affect children’s behavior as cyber bystanders, as well as how guidance within overprotection, such as setting boundaries while encouraging decision-making, can nurture a child’s ability to assess situations and take appropriate action. Longitudinal surveys are needed to identify the inflection points on how parental mediation, such as respecting children’s privacy and increasing monitoring, prompts future cyber bystander behavior. Such studies could identify key inflection points where specific types of parental involvement or mediation significantly impact children’s responses to cyberbullying.

Third, the findings of the current study showed that previous cyberbullying experiences can significantly influence bystander behavior. Some bystanders with prior victimization experiences may suffer in silence instead of defending themselves or other victims. Future studies may examine instances where such minimization occurs and consider its impact on bystanders’ willingness to intervene. They might also explore how bystanders use moral justification to reframe cyberbullying, viewing it as serving a socially acceptable or beneficial purpose, such as toughening up the victim or as a deserved social sanction. Schools should include curricula on identifying signs of emotional distress as part of their anti-bullying policies and encourage students to express their experiences to foster empathy. Additionally, role-playing scenarios can help students develop an understanding of a cyberbullying victim’s feelings and how various bystander responses might impact the victim. This nuanced understanding of the psychological barriers to bystander intervention in cyberbullying will help to identify potential areas for intervention and education, aimed at promoting more proactive bystander behavior in online spaces. Case studies and vignettes from existing interventions that have successfully engaged cyber bystanders and reduced cyberbullying incidents demonstrate their feasibility. Whole-school interventions targeting bystander behavior should be implemented to create a supportive environment and reduce the impact of these private forms of cyberbullying. Specifically, this may include guidelines for incorporating bystander intervention training into school curricula and best practices for educators to facilitate discussions about digital citizenship and empathy in classroom teaching. Peer mentoring programs that leverage the influence of social norms among adolescents, along with parental involvement in meaningful conversations with their children about cyberbullying, can reinforce the importance of being an active bystander at home. District policymakers may also develop clear anti-cyberbullying policies at both the school and district levels by creating collaborative partnerships between schools, parents, and community organizations to support cyberbullying prevention initiatives.

## Conclusion

In this study, we found that the differentiated impacts of cyberbullying victimization experiences on bystander reactions might be influenced by different parental rearing behaviors. Children raised by supportive parents often carry the warmth at home into their social interactions, potentially leading to more defensive reactions as cyber bystanders. The attitudes towards different victims could also be an essential element for behavior appraisal. Future research and interventions against cyberbullying may consider providing individualized training that includes enhancing parents’ positive parenting skills and fostering effective parent-child interactions. Moreover, there seems to be a lack of targeted parental interventions that address attitudes towards different victims and teach children communication literacy in an ethical manner.

## Data Availability

The datasets used and/or analysed during the current study are available from the corresponding author on reasonable request.
