# Correction: Reactions of adolescent cyber bystanders toward different victims of cyberbullying: the role of parental rearing behaviors

**DOI:** 10.1186/s40359-024-01913-4

**Published:** 2024-07-29

**Authors:** Qiqi Chen

**Affiliations:** Department of Applied Social Sciences, The Hong Kong Polytechnic University, Hong Kong, China

**Correction: BMC Psychology (2024) 12:377**

10.1186/s40359-024-01879-3

Following publication of the article, the author flagged that their given and family names were the wrong way around in the author list. The article has since been updated to correct this error, and the corrected author name may be seen in this erratum.

